# Tumour-Associated Neutrophils in Colorectal Cancer: a Systematic Review, Meta-analysis, and Comprehensive Literature Review

**DOI:** 10.1007/s12029-026-01517-8

**Published:** 2026-07-08

**Authors:** Ana Beatriz Kinupe Abrahao, Tiago Cordeiro Felismino, David Kerr

**Affiliations:** Department of Oncology, Medical Sciences Division, University of Oxford, Oxford, UK

**Keywords:** Colon cancer, Colorectal cancer, Tumour associated neutrophil, Tumour infiltrated neutrophil, Prognosis.

## Abstract

**Purpose:**

Neutrophils are at the forefront of innate immune response. The prognostic impact of tumour-associated neutrophils (TANs) remains unclear. This study aimed to evaluate the prognostic role of TANs in colorectal cancer (CRC).

**Methods:**

A systematic review and meta-analysis were conducted using PubMed, Embase, and Scopus to identify studies correlating TANs with time-to-event survival analysis in patients with stages I-IV CRC from the date of inception until April 2024. Articles were included if they involved the identification of TANs through hematoxylin-eosin (HE) or immunohistochemistry (IHC) and reported survival analysis. Data was collected from the tumour core (TC) and invasive margin (IM). The hazard ratios (HRs) were extracted from the study results. Heterogeneity was evaluated using Cochran’s Q and *I²*. Primary and secondary endpoints were overall survival (OS) and disease-free survival (DFS).

**Results:**

Of 4,292 citations found, 18 studies fulfilled eligibility criteria, encompassing 7,406 patients. IHC was used to identify TANs in 12 trials, while six performed HE. Five studies included patients with disease stages I-III, and 13 had stages I-IV. High TANs at the IM were associated with improved OS (HR = 0.64, 95% Confidence Interval [CI] 0.50–0.81, *I*^*2*^ = 48%) and DFS (HR = 0.47, 95% CI 0.25–0.88, *I*^*2*^ = 32%). At the TC, high TANs showed a trend towards better OS (HR = 0.82, 95% CI 0.61–1.10, *I*^*2*^ = 83%) and DFS (HR = 0.48, 95% CI 0.21–1.07, *I*^*2*^ = 93%), although not statistically significant and with high heterogeneity.

**Conclusion:**

In this systematic review and meta-analysis, high TANs at the invasive margin (IM), the tumour–host interface, was associated with improved prognosis in CRC. In contrast, no statistically significant association was observed for high TANs at the tumour core (TC).

## Introduction

Colorectal cancer (CRC) is one of the most frequently diagnosed malignancies worldwide and a leading cause of cancer-related mortality [1]. Tumour progression and metastatic dissemination in CRC are strongly influenced by the tumour microenvironment (TME), which contains diverse immune cell populations, including neutrophils, macrophages, dendritic cells, natural killer cells, and T and B lymphocytes [2–4].

Neutrophils, constituting the forefront of the host’s innate defense, comprise 50 to 70% of circulating leukocytes and can infiltrate diverse tissues through extravasation from the bloodstream. The neutrophil-to-lymphocyte ratio (NLR) is an emerging prognostic marker in CRC [5].

Despite their established role in innate immunity, the contribution of tumour-associated neutrophils (TANs) to cancer progression remains controversial. While some studies report anti-tumour functions of TANs—such as direct cytotoxicity against tumour cells and inhibition of metastatic spread—others describe pro-tumoural effects, highlighting the complexity and context-dependent nature of TAN biology [6–8].

TANs have been classically characterized by a dualistic state of activation and differentiation, in which N1 phenotypes are anti-tumorigenic, whereas N2 phenotypes are pro-tumorigenic [9]. N1-TANs have been associated with anti-tumour immune responses, including cytotoxic activity and activation of adaptive and innate immune cells, whereas N2-TANs have been linked to tumour progression through angiogenesis, extracellular matrix remodeling, and immunosuppressive interactions within the TME [10, 11]. Importantly, accumulating data suggest that TANs phenotypes demonstrate considerable functional plasticity and can represent a continuum rather than fixed categories [11, 12].

In addition to functional heterogeneity, the spatial distribution of TANs within tumours is variable. TANs located at the tumour core (TC), the central region of the tumour mass, are primarily involved in shaping the local TME. In contrast, TANs at the invasive margin (IM), the interface between the tumour and surrounding tissues, have been linked to angiogenesis and extracellular matrix remodelling, processes that may facilitate tumour invasion and dissemination [13, 14].

The biological significance of TANs is therefore highly context-dependent and influenced by intratumoral localization, cytokines, and microenvironmental factors such as hypoxia, inflammation, and elevated lactic acid levels [15]. In this context, previous studies have suggested that TANs at the invasive margin are more likely to display an antitumour, N1 phenotype [16].

Given the conflicting evidence regarding the prognostic implications of TANs, their clinical relevance remains incompletely defined. This systematic review and meta-analysis aim to evaluate the association between TANs and prognosis in patients with CRC.

## Materials and Methods

### Literature Search Strategy

A systematic review was conducted using PubMed, Embase (OVID version), and Scopus (OVID version) databases to identify studies correlating TANs with time-to-event survival analysis in patients with stages I-IV CRC from the date of their inception until April 2024. “Colorectal cancer” AND “tumour-associated neutrophils” OR “tumour-infiltrating neutrophils” AND “prognosis” were searched. All relevant key variations were used for these terms. This systematic review followed the recommendations of the Preferred Reporting Items for Systematic Reviews and Meta-Analyses (PRISMA) [17]. A comprehensive list of all search strings, Boolean operators, and MeSH/Emtree terms used for each database is provided in Table S1 (supplementary material).

### Study Selection Criteria

All studies were reviewed initially based on the title and abstract by a single reviewer. The full-text article was reviewed if the data was insufficient based on the title and abstract. Articles were included if they were published in the English language, performed on adult human patients, included CRC patients regardless of disease stage, involved the identification of TANs through hematoxylin-eosin (HE) staining or immunohistochemistry (IHC) staining techniques and at least one of the outcomes of interest (time-to-event survival analysis) were reported. Both retrospective and prospective studies were eligible for inclusion. Only the most recent publication was selected for inclusion if multiple publications were available for the same study.

Key exclusion criteria were as follows: studies that did not include a time-to-event survival analysis, trials involving immunotherapy drugs, only in vitro experiments, or animal models. Studies primarily focused on blood neutrophils rather than TANs, studies based only on gene expression analysis to characterize neutrophils, and studies that combined TANs with other biomarkers for outcome measures (and only reported these combined results) were excluded. Additionally, analyses exclusively involving public databases were excluded.

Time-to-event analyses included the following endpoints: overall survival (OS), defined as the time between cancer diagnosis (or randomization, in case of an interventional clinical trial) and death from any cause (with patients alive or lost to follow-up being censored); disease-free survival (DFS), defined as the time from the end of curative cancer treatment until recurrence of the disease or death; recurrence-free survival (RFS), defined as the time between the end of curative cancer treatment and cancer recurrence or relapse.

### Outcomes

The primary endpoint of interest was OS. The secondary endpoint was DFS. For the purpose of this meta-analysis, DFS and RFS were aggregated and analyzed as a single endpoint.

### Synthesizing the Evidence

The hazard ratios (HR) with 95% confidence interval (CI) were directly extracted from the study results corresponding to the predefined outcomes of interest. Preference was given to HRs with 95% CIs obtained from multivariable analyses. However, in the absence of such data, HRs were extracted from univariable analyses. For studies lacking HR data, HR was calculated based on the total number of subjects per cohort, the number of events per cohort, and the provided p-values [18].

For the meta-analysis, low TANs were used as the reference category (HR = 1). In instances where the original studies reported high TANs as the reference group, HRs and their 95% CIs were recalculated to align with the reference category. Specifically, the HR for this meta-analysis was calculated as the inverse of the reported HR (HR_meta = 1/HR_reported). Similarly, the lower bound of the 95% CI for the meta-analysis was recalculated as the inverse of the upper bound of the reported 95% CI (Lower_95%_CI_meta = 1/Upper_95%_CI_reported), and the upper bound of the 95% CI for the meta-analysis was recalculated as the inverse of the lower bound of the reported 95% CI (Upper_95%_CI_meta = 1/Lower_95%_CI_reported).

Data on TANs were collected from both the TC and the IM. In instances where the study did not specify the precise tumour region from which the TANs were analyzed, it was assumed that the data corresponded to the tumour core. The cutoff data concerning TANs values were also extracted. When the study provided multiple possible cutoff values, the lowest value was compared to the highest value to calculate and extract the HR 95% CI.

To synthesize data from the included trials, an aggregated table was built comparing clinical and pathological characteristics between low and high TANs groups. This table included important demographic and clinicopathological variables that were available in the publications (sex, tumour grade differentiation, primary tumour location, sidedness, disease staging and microsatellite status. Data were extracted and compiled for each trial into the table, with totals calculated for each characteristic across all trials.

### Statistical Analysis

EndNote (Version 20.4) was used for the systematic review step. Statistical analyses were performed using R and RStudio (Version 2023.12). The ‘meta’ package in R was utilized to conduct the meta-analysis. Heterogeneity among the included studies was evaluated using Cochran’s Q and *I²* statistics. For cases where *I²* exceeded 50%, the Mantel-Haenszel random effects model was employed to pool the data; otherwise, the Inverse-Variance fixed effects model was applied. The DerSimonian and Laird method was employed to estimate the heterogeneity among studies (applied to the random effects model). Chi-square tests were performed to determine the statistical significance of differences between demographic and clinicopathological characteristics (categorical variables) across high and low TANs groups.

Egger’s and Begg’s tests were employed to assess funnel plot asymmetry objectively. Egger’s test uses a regression approach to detect funnel plot asymmetry, while Begg’s test assesses asymmetry through rank correlation.

All statistical tests were two-sided, and a p-value of less than 0.05 was considered to indicate statistical significance.

This systematic review and meta-analysis used only previously published data and involved no new human participants; therefore, ethics approval and informed consent were not required. All original studies were conducted in accordance with institutional and national ethical standards and the Declaration of Helsinki.

### Study Quality

The quality of the studies included in this meta-analysis was assessed using the Newcastle-Ottawa Scale (NOS) [19], a tool specifically designed for evaluating the quality of nonrandomized studies.

The NOS evaluates studies based on three basic criteria: selection, comparability, and outcome (or exposure). The selection criterion analyzes the representativeness of the study participants and the adequacy of case definitions. The comparability criterion evaluates the similarity of study groups based on design or analysis, ensuring that relevant factors are controlled. The outcome (or exposure) criterion examines the measurement of outcomes and adequate follow-up. Each study is awarded stars for meeting quality items within these criteria, with a maximum of nine stars indicating the highest quality.

## Results

### Literature Review Results

**Fig. 1:**
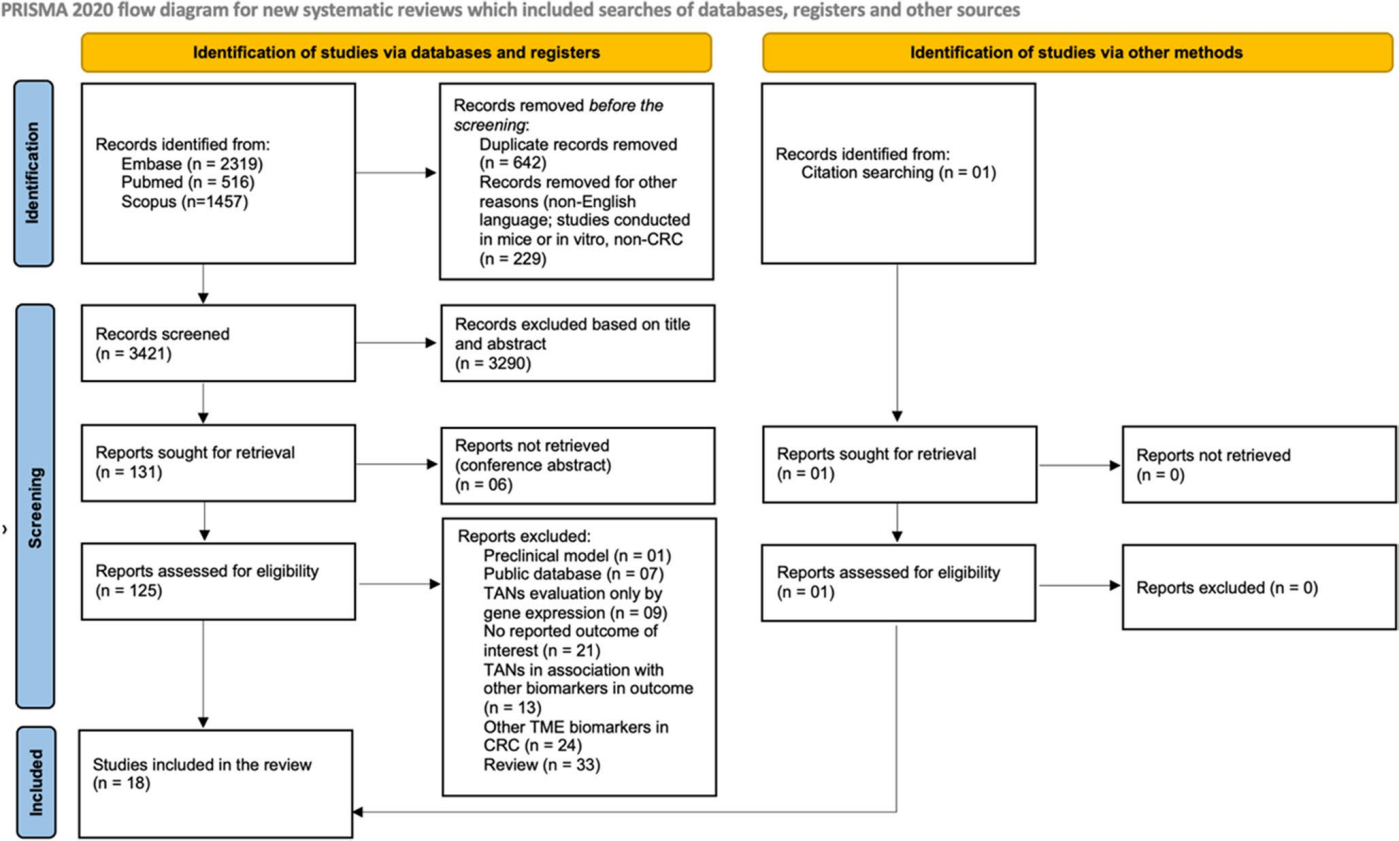
Study Selection using PRISMA 2020 Flow Chart Diagram

Comprehensive database searches identified 4292 citations (Fig. 1). During the initial analysis, 642 duplicate abstracts were excluded, as were 229 studies that did not meet the inclusion criteria due to non-English language publications, studies conducted on mice or in vitro, or studies unrelated to colorectal cancer. All 3421 remaining abstracts were reviewed, and 18 studies fulfilled eligibility criteria. These selected studies reported at least one outcome of interest.

### Study Design of Included Trials

Table S2 (supplementary material) presents the primary characteristics of 18 eligible trials encompassing 7,406 patients. Notably, none of the studies exclusively focused on colon or rectal cancers. Geographically, the majority of the studies were conducted in Europe (K = 8) [13, 20–26], followed by Asia (K = 7) [27–33] and North America (K = 3) [34–36]. The sample sizes of the included studies ranged from 84 to 1,225 patients. Follow-up durations varied between 57.4 and 68 months; however, seven studies did not report follow-up times [25, 26, 29, 30, 32, 33, 35].

Two studies analyzed prospective cohorts [20, 24]. Berry et al. [34] selected tumour biopsies retrospectively; however, patient information was collected prospectively. All remaining studies were retrospective in nature.

Although the majority of the studies investigated patients with stages I to IV, five studies included only patients with disease stages I to III [21, 25, 30–32]. Berry et al. [34] did not explicitly report the tumour biopsy region as a TC; however, the description provided for TANs suggested that the tumour region was TC. Similarly, Zhang et al. [33]. did not specify whether the tumour biopsy was from TC or the IM. Nonetheless, the description indicated that 87 out of 93 patients had biopsies from both TC and IM. Since these tumour locations were not separated in the results, we have assumed them to be TC. Additionally, two studies did not report the tumour region from which the biopsy was performed, we assumed them to be TC [29, 30].

Twelve trials employed IHC to detect TANs, with various markers being assessed. Eight trials identified TANs using the CD66b^+^ marker [23, 25–27, 29–32], while myeloperoxidase (MPO+) staining was utilized in two trials [22, 33]. Additionally, the CD177^+^ marker was used in one trial [28], and neutrophil elastase staining was applied in another trial [24]. In contrast, six studies employed HE (morphology) as a detection method for TANs [13, 20, 21, 34–36].

The cutoff values for TANs to analyze outcomes of interest varied across the trials. Six studies used the median as the cutoff reference for TANs [21, 23, 28–30, 34]. Three studies employed the Receiver Operating Characteristic (ROC) curve as a reference [24, 27, 32]. Two studies determined TANs cutoff values using regression tree analysis [22, 25]. Two studies categorized TANs as absent or weak (or none and sporadic) versus moderate or severe (or moderate and abundant) for their cutoff data [13, 26]. Vayrynen et al. [35] analyzed TANs in quartiles (C1 to C4); this meta-analysis utilized the comparison between C1 and C4 as a cutoff. Nielsen et al. [20]. employed four strata for TANs (S1 to S4), although the authors did not specify if these strata were equally divided into quartiles; for this meta-analysis, the comparison between S1 and S4 was adopted. Rottmann et al. [36] categorized TANs as low plus intermediate versus high as a cutoff value. Xu et al. [31] utilized the StepMiner algorithm [37] for TANs cutoff value.

### Tumour-Associated Neutrophils and CRC Prognosis

**Fig. 2:**
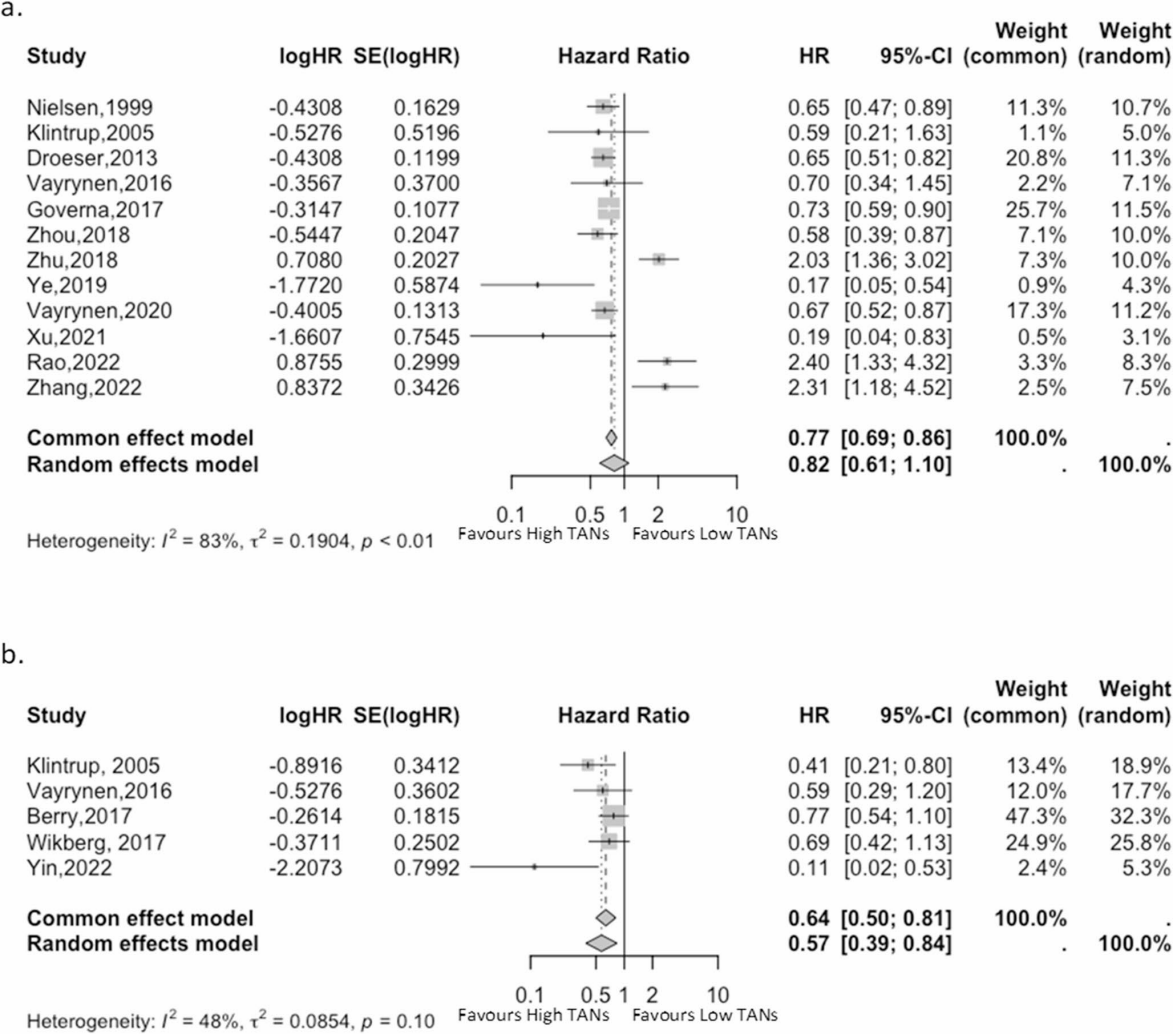
Forest plots of the prognostic association of tumour-associated neutrophils in relation to overall survival. (**a**) Tumour Core (**b**) Invasive Margin

A total of twelve studies reported on the prognostic impact of TANs identified at the TC in relation to OS (Fig. 2a). The heterogeneity among these studies was high (*I²* = 83%); thus, a random effects model was employed, yielding a HR of 0.82 (95% CI 0.61–1.10). Five studies reported the prognostic impact of TANs at the IM in relation to OS (Fig. 2b). In this analysis, heterogeneity was low (*I²*= 48%), allowing for using a common effects model. The HR was 0.64 (95% CI 0.50–0.81), indicating that the high TANs group have a better prognosis.

**Fig. 3:**
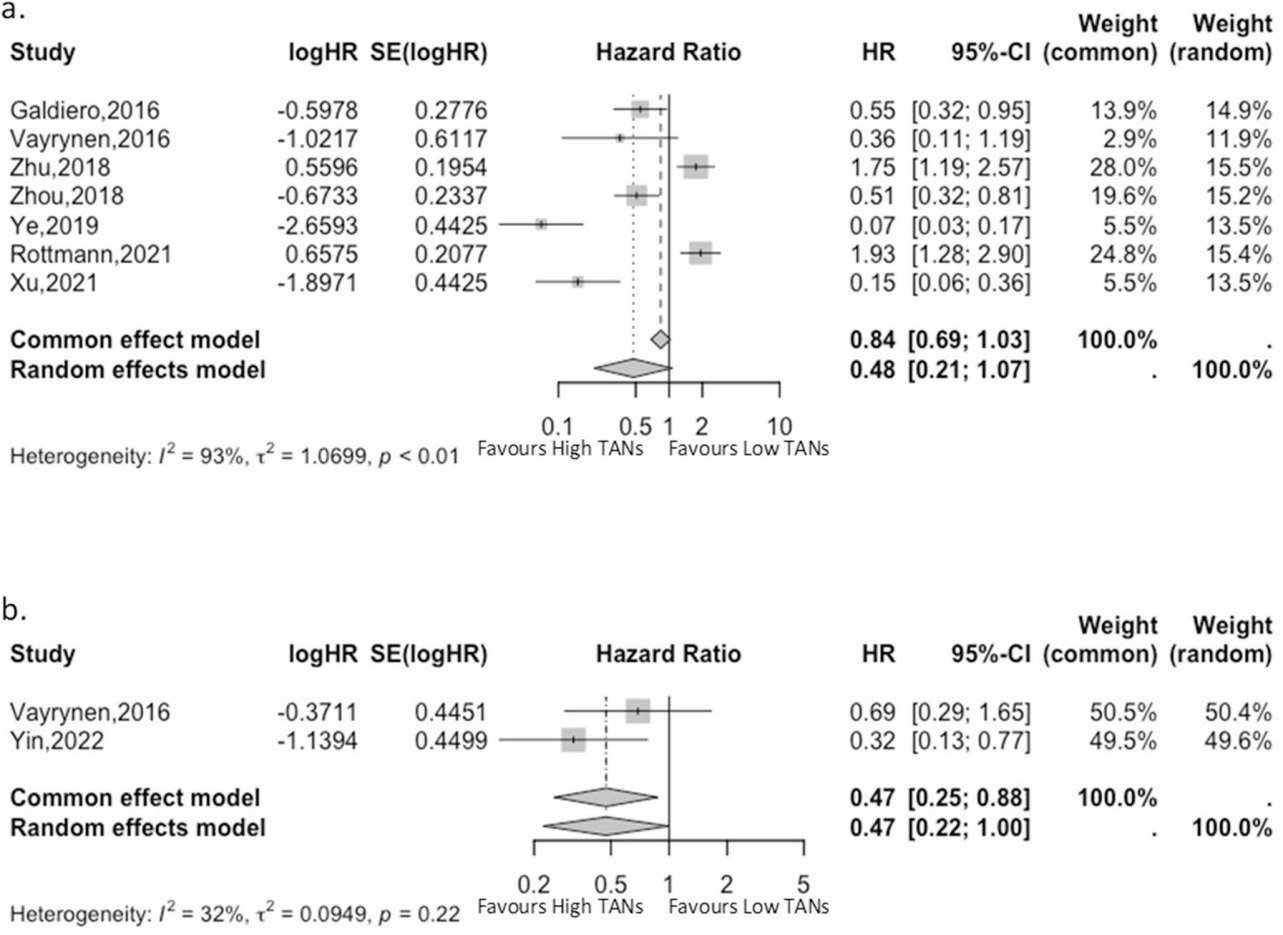
Forest plots of the prognostic association of tumour-associated neutrophils in relation to disease-free survival. (**a**) Tumour Core (**b**) Invasive Margin

Seven trials investigated the prognostic impact of TANs at the TC in relation to DFS (Fig. 3a). This analysis exhibited high heterogeneity (*I²* = 93%). Using a random effects model, the HR was 0.48 (95% CI 0.21–1.07). Additionally, two studies examined the prognosis of TANs at the IM in relation to DFS (Fig. 3b). The heterogeneity in this analysis was low (*I²* = 32%). Employing a common effects model, the HR was 0.47 (95% CI 0.25–0.88).

Overall, the data suggest that high TANs infiltration at the IM is associated with a better prognosis in CRC patients. The analysis regarding the prognostic effect of TANs at the TC indicated high heterogeneity among studies and did not demonstrate a statistically significant benefit of high TANs.

A single trial conducted by Richards et al. [21] analyzed TANs at the IM and used cancer-specific survival as the time-to-event outcome. The analysis revealed a trend suggesting a beneficial relationship between high TANs at the IM and CSS. However, this relationship was not statistically significant, with an HR of 0.68 (95% CI: 0.35–1.33) and a p-value of 0.21.

### Association of Clinical and Pathological Characteristics and TANs

A detailed evaluation of clinical subgroups was conducted to investigate the correlation of TANs with sex, tumour grade (low versus moderate/high-grade), primary tumour location (colon versus rectum), tumour sidedness (right versus left-sided), tumour stage (I-III versus IV), and the microsatellite status (microsatellite instability-high versus microsatellite stable). These analyses were based predominantly on TANs data from the TC, as most studies did not stratify findings by tumour region. The results are summarized in Table 1.

**Table 1:** Association of Clinical and Pathological Characteristics and tumour-associated neutrophils (TANs) levels

Clinical Characteristics (*n*)	Trials	Low TANs (%)	High TANs (%)	p*
Sex (*n* = 3,024)				
Female	8 [22, 27, 28, 31, 34, 36, 38, 39]	704 (49%)	648 (45%)	0.86
Male		864 (51%)	808 (55%)	
Tumour Grade (*n* = 2,365)				
Low grade	5 [27, 28, 31, 36, 38]	564 (32%)	203 (35%)	0.21
Moderate/high-grade		1,215 (68%)	383 (65%)	
Tumour Location (*n* = 2,410)				
Colon	5 [27, 28, 31, 38, 39]	968 (58%)	440 (60%)	0.37
Rectum		707 (42%)	295 (40%)	
Sidedness (*n* = 768)				
Right-sided	3 [34, 36, 38]	232 (38%)	121 (47%)	0.02
Left-sided		378 (62%)	137 (53%)	
Disease Stage (*n* = 2,023)				
Early Stage (I-III)	6 [22, 27, 28, 34, 36, 38]	1,164 (86%)	588 (89%)	0.08
Metastatic (IV)		195 (14%)	76 (11%)	
Microsatellite Status (*n* = 1,987)				
Microsatellite Instability – high (MSI-H)	3 [22, 36, 38]	284 (17%)	82 (23%)	0.03
Microsatellite Stable (MSS)		1,340 (83%)	281 (77%)	

* Chi-square test

Eight studies investigated the relationship between TANs and sex, encompassing a total of 3,204 patients [22, 26–28, 30, 31, 34, 36]. Within the low TANs group, 49% of the patients were female, while 51% were male. In the high TANs group, 45% of the patients were female, and 55% were male. Statistical analysis revealed no significant differences in sex distribution between low and high TANs groups (*p* = 0.86).

Five studies provided data on the relationship between TANs and tumour grade, including 2,365 patients [26–28, 31, 36]. In the low TANs group, 32% of the patients had low-grade tumours, while 68% had moderate/high-grade tumours. In the high TANs group, 35% of the patients had low-grade tumours, whereas 65% had moderate/high-grade tumours. Statistical analysis revealed no significant differences between tumor grade and TANs groups (*p* = 0.21).

Additionally, five studies analyzed the association between primary tumour location (colon versus rectum) and TANs groups, involving 2,410 individuals [26–28, 30, 31]. In the low TANs group, 58% of patients had colon cancer, while 42% had rectal cancer. In the high TANs group, 60% of patients had colon cancer, and 40% had rectal cancer. Statistical analysis revealed no significant differences between primary tumour location and TANs groups (*p* = 0.37).

Three trials provided data on tumour sidedness and TANs groups, encompassing 768 patients [26, 34, 36]. In the low TANs group, 38% of patients had right-sided CRC, while 62% had left-sided CRC. In the high TANs group, 47% of patients had right-sided CRC, and 53% had left-sided CRC. Statistical analysis demonstrated a significant association between tumour-sidedness and TAN levels (*p* = 0.02), indicating a higher proportion of left-sided CRC in the low TANs group.

Regarding disease staging, six trials evaluated the relationship between TANs groups and disease stages, encompassing a total of 2,023 patients [22, 26–28, 34, 36]. In the low TANs group, 86% of patients had early-stage disease (stages I to III), while 14% had metastatic disease (stage IV). In the high TANs group, 89% of patients had early-stage disease, and 11% had metastatic disease. Statistical analysis revealed no significant relationship between disease stage and TANs levels (*p* = 0.08).

Three studies, including 1,987 patients, analyzed microsatellite status and TANs groups [22, 26, 36]. In the low TANs group, 17% of patients had microsatellite instability-high (MSI-H), while 83% were microsatellite stable (MSS). In the high TANs group, 23% of patients were MSI-H, and 77% were MSS. Statistical analysis indicated a significant association between microsatellite status and TANs levels (*p* = 0.03), with a higher proportion of MSI-H observed in the high TANs group.

### Quality of Included Trials

The quality of the included studies was evaluated using the NOS, with scores ranging from five to eight in the included trials, as presented in Table S3 (supplementary material). The results indicate that one study received a score of five, five studies received a score of six, five studies received a score of seven, and seven studies received a score of eight. Consequently, 12 out of the 18 included studies achieved a score of seven or higher, signifying that most of the included studies were of good quality [40, 41].

### Publication Bias Effect Analyses

In the analysis of TANs at the TC and OS (K = 12), both Egger’s test (*p* = 0.7737) and Begg’s test (*p* = 0.5833) showed no evidence of asymmetry. In the analysis of TANs at the IM with OS (K = 5), Egger’s test was statistically significant (*p* = 0.0159), while Begg’s test showed a borderline result (*p* = 0.05). In the analysis of TANs at the TC with DFS (K = 7), Egger’s test was statistically significant (*p* = 0.03), whereas Begg’s test was not (*p* = 0.22). For TANs at the IM with DFS, only two studies were included (K = 2); therefore, formal assessment of publication bias was not performed.

## Discussion

In this systematic review and meta-analysis, 18 eligible trials were included, encompassing 7,406 patients in which TANs were analyzed as a prognostic marker in CRC. To the best of current knowledge, this is the largest meta-analysis performed to answer this particular question in a single tumour type. Importantly, a validated metric, the NOS, was employed to assess the quality of the included studies. The results indicated that 12 out of the 18 studies achieved a score of seven or higher, implying that the majority of the studies were of good quality.

A descriptive analysis of the main clinicopathological characteristics across low TANs versus high TANs groups was conducted using data available in the published studies. The analysis included sex, tumour grade differentiation (low versus moderate/high-grade tumours), primary tumour location (colon versus rectum), tumour sidedness (right versus left-sided), staging (early-stage versus metastatic), and microsatellite status (MSS versus MSI-H).

Notably, a statistically significantly higher proportion of left-sided tumours was observed among cases with low TANs (*p* = 0.02). Conversely, a higher proportion of MSI-H tumours was found among patients in the high TANs group (*p* = 0.03).

This research demonstrated that high TANs at the IM are associated with better prognosis in patients with CRC, both in the OS analysis (HR = 0.64, 95% CI 0.50–0.81) and DFS analysis (HR = 0.47, 95% CI 0.25–0.88). Conversely, no statistically significant association was identified between TANs at the TC and OS (HR = 0.82, 95% CI 0.61–1.10) or DFS (HR = 0.48, 95% CI 0.21–1.07).

The hypothesis that TANs are influenced by local factors within distinct regions of the tumours may help elucidate the different impacts in prognosis related to neutrophil localization (e.g., TC versus IM) [42]. Chengzeng et al. [43] demonstrated a significant association between decreased expression of CD66b^+^ TANs in the tumour margin in patients with stage III CRC (*p* = 0.05), a relevant poor prognostic factor in non-metastatic patients. In addition, the authors suggested that a lower density of CD66b^+^ TANs at the IM could be related to immune escape.

The TC and IM are two distinct regions [44]. TC is commonly defined as the central part of the tumour mass, typically consisting of the bulk of the tumour cells. Conversely, IM refers to a region centered on the border separating the host tissue from malignant cells [45]. These two regions differ significantly in cell composition [46, 47], blood vessel presence [48, 49], oxygen and nutrient supply [50, 51], and potentially the molecular characteristics of tumour cell subpopulations [52].

Kather et al. [53] demonstrated that densities of tumour-infiltrating lymphocytes (TILs), described as cytotoxic CD8^+^ T cells, and CD163^+^ macrophages were higher at the outer invasive margin (outer 500 μm) [53]. TANs were also found to be higher at the IM in comparison to the TC among different tumour types [54, 55].

TANs at the IM may hold prognostic value complementary to established immune-based classifiers in CRC. Given their spatial proximity to the tumor–stroma interface, TANs at the IM could refine prognostic stratification by capturing dynamic interactions between the tumor and immune microenvironment. Integrating TANs density or phenotype into existing immune-score frameworks may improve prediction of recurrence risk and survival beyond conventional clinicopathologic variables. Such incorporation could ultimately support personalized therapeutic decision-making, particularly in identifying patients who might benefit from intensified adjuvant therapy or immunomodulatory approaches [56–59].

However, the literature regarding the abundance of TANs across the TC and IM in CRC remains inconclusive. Wikberg et al. [26] reported that in the IM (referred to as the tumour front in the publication), 62.1% of cases exhibited no or scarce CD66b^+^ neutrophil infiltration, 15.6% showed modest density of CD66b^+^ cells, 12.6% demonstrated abundant infiltration, and 9.8% exhibited highly abundant infiltration. At the TC, the respective numbers were 49.9%, 23.0%, 16.6% and 10.5%. Overall, these numbers suggest a higher infiltration of TANs at the TC. However, due to the paucity of data, this question is yet to be answered.

Blood supply, oxygen, and nutrient availability are also heterogeneous across different regions of the tumour [60, 61]. In an oversimplistic approach, IM tends to have a higher blood supply, which results in higher availability of oxygen and nutrients, such as important amino acids [62, 63]. These characteristics may influence immune cells [63], which ultimately can modulate their phenotype into a more pro- or anti-tumoral profile.

Interestingly, neutrophils rely heavily on glucose levels for adenosine triphosphate (ATP) production through glycolysis [64, 65], as their mitochondria do not contribute to ATP synthesis [66]. Cancer cells are also highly dependent on glycolysis, even in the presence of oxygen, as described by Warburg et al. [67]. Previous research demonstrated that glucose competition has been associated with cancer progression [68, 69]. In summary, environmental conditions may impair the normal functions of TANs and drive changes in their phenotypic states.

The IM is recognized as a critical site where tumour cells interact with immune cells [53, 70]. For instance, in CRC, the density of CD8^+^ T lymphocytes at the IM is well-established as an important prognostic marker [71]. It is plausible that TANs at the IM also play an important role in cancer control, although further research is essential to test this hypothesis.

In addition to the specific metabolic conditions, the patterns of chemokines, cytokines, and immune cell interactions can provide valuable insights into the prognostic effects of TANs. For instance, in the presence of TGF-β, TANs tend to have an N2 profile, which is associated with pro-tumoral properties. In the presence of interferon beta (IFNβ) or inhibition of TGF-β, TANs switch to an N1 phenotype, which is anti-tumoral [72, 73]. Furthermore, when TGF-β activity is blocked, TANs become cytotoxic and activate CD8^+^ T cells[74].

Interestingly, TGF-β has an important prognostic role in CRC. Multiple studies have demonstrated that higher levels of TGF-β are related to cancer progression through various mechanisms [75, 76]. Moreover, TGF-β is a marker of Consensus Molecular Subtype 4 (CMS4) CRC, which is characterized by stromal invasion, angiogenesis and poor prognosis [77].

The interactions between TANs and TILs are also important to understand mechanisms regulating the immune response. Erulasnov et al. [78]. showed that TANs represent a significant part of immune cells in TME of resected lung cancer. Moreover, they observed that TANs exhibit high phagocytic activity and the ability to generate reactive oxygen species. They also showed that TANs released various immunoregulatory cytokines, chemokines, and growth factors. Finally, the authors demonstrated that TANs increased T cell interferon-gamma production and activation and amplified T cell proliferation [78]. Kousis et al. [79] also observed that neutrophils have a direct role in T-cell proliferation following photodynamic therapy in tumour-bearing mice (Colo26 - murine colon carcinoma) [80].

Nevertheless, many studies have identified TANs as immunosuppressive and associated with poorer prognosis. A meta-analysis published in 2014 [81], evaluated the prognostic impact of TANs in various tumours. The authors included 20 articles with a total number of 3946 patients. Importantly, two studies involved CRC patients, but a subgroup analysis of CRC was not performed. They divided the analysis into intratumoral, peritumoral and stromal TANs. The pooled HR of intratumoral TANs was 1.66 (95% CI 1.37–2.01, *I*^*2*^= 70.5%), indicating that higher densities of intratumoral TANs were independently associated with poor prognosis.

Interestingly, the prognostic impact of specific immune cells at the TME may vary according to the type of tumour. Forkhead box P3 (FOXP3^+^) regulatory T cells are a classical example[82]. While they are associated with poor prognosis in a range of different tumours [82, 83], they might be associated with a better prognosis in CRC [84]. Therefore, it is premature to draw definitive conclusions about the role of TANs across different tumour types.

In this systematic review and meta-analysis, a detailed evaluation of subgroups was conducted to investigate the correlation of TANs with relevant demographics and clinicopathological subgroups. This analysis has limitations as studies have diverse methodologies. Nonetheless, it was demonstrated that in the high TANs group, 23% of tumours were MSI-H, while in the low TANs groups, 17% were MSI-H (*p* = 0.03). There is a large body of evidence that relates MSI-H tumours with better prognosis, particularly in the nonmetastatic setting [85, 86]. Moreover, MSI-H tumours are known for high TILs infiltration, which is also related to better survival [57]. However, due to limited data, it was not possible to perform survival subgroup analysis according to microsatellite status.

It was also observed that in the high TANs group, 47% of the tumours were right-sided, while in the low TANs group, 38% were right-sided (*p* = 0.02). Primary tumour sidedness has been implicated in specific clinicopathological characteristics [87] and also in prognosis, particularly in the metastatic setting [88, 89]. From the CMS classification, CMS1 is enriched by right-sided, MSI-H, and BRAF V600E mutated tumours [90]. In metastatic patients, both CMS1 and right-sided tumours are linked to a worse prognosis. However, in early-stage disease, the prognostic impact is less clear [77]. A survival subgroup analysis was also not performed due to limitations.

A recent meta-analysis by Jiang et al. [91] systematically evaluated the prognostic role of TANs in CRC and reported that high TANs in the peritumoral compartment were associated with improved cancer-specific survival, while no significant associations were observed for OS or DFS. More recently, Yang et al. [92] performed an updated meta-analysis including 25 studies and 10,356 patients and demonstrated that high TAN infiltration was associated with improved cancer-specific survival, whereas associations with OS and DFS appeared more heterogeneous and dependent on tumour stage and methodological factors.

Despite the larger sample sizes of these prior analyses, our study provides complementary information due to important methodological differences. First, we applied stricter histopathological criteria for TAN identification, restricting inclusion to studies using HE or IHC, while excluding gene-expression and database-derived neutrophil estimates or transcriptomic surrogates. Second, we performed a refined spatial analysis by separately evaluating TANs at the TC and IM, rather than grouping all peritumoral regions together. Finally, our eligibility criteria included patients across all disease stages (I–IV). These methodological differences may partly explain discrepancies across studies and support the complementary relevance of our findings.

This study has several limitations. It includes studies spanning more than two decades, during which methodological approaches have evolved from manual assessment to digital pathology and machine-learning techniques. Additionally, some hazard ratios were indirectly derived, which may have introduced variability and reduced precision. Finally, the literature search was conducted up to April 2024, and more recent studies may not have been captured.

Moreover, the identification of TANs largely varied across the studies. Some authors decided to classify TANs only based on HE (morphology), while others have used different IHC markers. This topic has been debated in the literature. There is no agreement on what would be the most accurate method to detect TANs [93]. For instance, CD66b^+^, which was frequently used in studies included in this systematic review, can also be expressed by eosinophils [94, 95]. Furthermore, different markers can be detected in different stages of maturation of neutrophils and can be expressed or not according to stimulation from different cytokines and chemokines [96]. For example, CD177^+^ is expressed in mature neutrophils but can also be detected in less mature myelocytes [97]. In addition, MPO^+^ can also be detected in monocytes and macrophages [98].

The heterogeneity in TANs identification likely reflects the use of distinct markers that capture different neutrophil subsets or activation states. For instance, CD66b and CD177 are associated with mature and activated neutrophils, whereas MPO may represent a broader spectrum of resting or degranulating phenotypes. These differences may account for the divergent prognostic associations observed across studies, emphasizing the need for standardized and functionally informed approaches to TANs characterization [59, 99–101].

In addition, studies based on transcriptomic analyses were excluded. mRNA expression profiling has gained increasing attention in recent years. This methodology has the potential to identify different clusters of cells [102] and can describe different activation states in cells (from inactivated to highly active) [103]. Furthermore, single-cell analysis can accurately differentiate groups of cells in a highly intricate and complex TME [104]. However, these techniques have not been incorporated in clinics yet and cannot be used for decision-making in clinical practice.

This study also faces inherent heterogeneity across the included cohorts. Differences in patient stage distribution, TANs detection methods (HE versus IHC), and cutoff definitions for high versus low TANs density represent major sources of variability and limit the comparability of results. Moreover, the assumption that TANs data originated from the tumour core when the specific region was not reported may have introduced non-differential misclassification, given that the immune microenvironment can differ markedly between the tumour core and invasive margin.

The absence of standardized cutoff values—ranging from median-based to quartile-based thresholds—further contributes to inconsistency across studies. Collectively, these factors constitute important constraints of the pooled analysis and highlight the need for standardized methodologies in future research.

Although Egger’s and Begg’s tests were applied to assess potential publication bias, the results should be interpreted with caution due to the small number of studies included in certain analyses (K < 10). Both tests have limited power in small subsets and may yield inconsistent results, particularly when between-study heterogeneity is present. Therefore, any apparent asymmetry or discrepancy between these tests may not reliably reflect true publication bias, underscoring the need for cautious interpretation and complementary qualitative assessment.

Finally, evaluating the prognostic impact of a single cell type within the complex TME may be imprecise. Immune cells and cytokines exhibit dynamic interactions with overlapping functions. These interactions can vary spatially within the tumour (TC versus IM) and by tumour location within the body (primary versus metastatic site), and they may change over time due to treatment pressures.

## Conclusion

This systematic review and meta-analysis demonstrates that high TANs at the IM is associated with improved OS and DFS in CRC. These findings underscore the importance of spatial immune contexture and support future studies using standardized, spatially resolved and integrative approaches to better define the prognostic role of TANs.

## Data Availability

The datasets generated during and/or analysed during the current study are available from the corresponding author on reasonable request.
